# *Rubia cordifolia* L. Attenuates Diabetic Neuropathy by Inhibiting Apoptosis and Oxidative Stress in Rats

**DOI:** 10.3390/ph16111586

**Published:** 2023-11-09

**Authors:** Sweeti Bana, Nitin Kumar, Ali Sartaj, Abdulsalam Alhalmi, Ashraf Ahmed Qurtam, Fahd A. Nasr, Mohammed Al-Zharani, Neelam Singh, Praveen Gaur, Rosaline Mishra, Snigdha Bhardwaj, Hasan Ali, Radha Goel

**Affiliations:** 1 Department of Pharmacology, Lloyd School of Pharmacy, Greater Noida 201306, India; sweetybanapharm8755@gmail.com; 2 Department of Pharmacy, Meerut Institute of Technology, Meerut 250103, India; nitin_23106@yahoo.co.in (N.K.); hasanmesra@gmail.com (H.A.); 3 Department of Pharmaceutics, Lloyd School of Pharmacy, Greater Noida 201306, India; sartaz005@yahoo.com; 4 Department of Pharmaceutics, School of Pharmaceutical Education and Research, Jamia Hamdard, New Delhi 110062, India; asalamahmed5@gmail.com; 5 Department of Biology, College of Science, Imam Mohammad Ibn Saud Islamic University (IMSIU), Riyadh 11623, Saudi Arabia; aaqurtam@imamu.edu.sa (A.A.Q.); faamohammed@imamu.edu.sa (F.A.N.); mmyalzahrani@imamu.edu.sa (M.A.-Z.); 6 Department of Pharmacy, ITS College of Pharmacy, Muradnagar 201206, India; singhneelam16@gmail.com; 7 Department of Pharmacy, Metro College of Health Sciences and Research, Plot No.-41, Knowledge Park-III, Uttar Pradesh 201306, India; gaurpharm@gmail.com (P.G.); rosalinemishra.mchsr@gmail.com (R.M.); 8 Department of Pharmacy, Noida Institute of Engineering and Technology, Greater Noida 201306, India; snigs1990@gmail.com; 9 Department of Pharmacology, Lloyd Institute of Management & Technology, Plot No.-11, Knowledge Park-II, Greater Noida 201306, India

**Keywords:** antioxidants, antidiabetic, diabetic neuropathy, *Rubia cordifolia*, streptozotocin, caspase-3, Bax:Bcl2 ratio, brain tissue, sciatic nerve tissue

## Abstract

Background: Diabetic neuropathy is a debilitating manifestation of long-term diabetes mellitus. The present study explored the effects of the roots of *Rubia cordifolia* L. (*R. cordifolia* L.) in the Wistar rat model for diabetic neuropathy and possible neuroprotective, antidiabetic, and analgesic mechanisms underlying this effect. Materials and Methods: Rats were divided into five experimental groups. An amount of 0.25% carboxy methyl cellulose (CMC) in saline and streptozotocin (STZ) (60 mg/kg) was given to group 1 and group 2, respectively. Group 3 was treated with STZ and glibenclamide simultaneously while groups 4 and 5 were simultaneously treated with STZ and hydroalcoholic extract of the root of *R. cordifolia*, respectively. Hot plate and cold allodynias were used to evaluate the pain threshold. The antioxidant effects of *R. cordifolia* were assessed by measuring Thiobarbituric acid reactive substances (TBARS), reduced glutathione (GSH), catalase (CAT), and superoxide dismutase (SOD). At the end of the study, sciatic nerve and brain tissues were collected for histopathological study. Bcl-2 proteins, cleaved caspase-3, and Bax were assessed through the Western blot method. Results: *R. cordifolia* significantly attenuated paw withdrawal and tail flick latency in diabetic neuropathic rats. *R. cordifolia* significantly (*p* < 0.01) improved the levels of oxidative stress. It was found to decrease blood glucose levels and to increase animal weight in *R. cordifolia*-treated groups. Treatment with *R. cordifolia* suppressed the cleaved caspase-3 and reduced the Bax:Bcl2 ratio in sciatic nerve and brain tissue compared to the diabetic group. Histopathological analysis also revealed a marked improvement in architecture and loss of axons in brain and sciatic nerve tissues at a higher dose of *R. cordifolia* (400 mg/kg). Conclusion: *R. cordifolia* attenuated diabetic neuropathy through its antidiabetic and analgesic properties by ameliorating apoptosis and oxidative stress.

## 1. Introduction

Diabetic neuropathy is an enervating manifestation that arises in patients with long-term diabetes mellitus [1]. As per a recent study, approximately 12% of patients with diabetes mellitus showed the presence of diabetic neuropathy. Diabetic neuropathy causes functional abnormalities, disabilities, and peripheral nerve damage to the organ affected by prolonged-term diabetes [2,3]. Diabetic neuropathy is heterogeneous, affecting multiple parts of the nervous system with symptoms such as numbness, burning sensations, and sharp pains in the different body parts [4,5,6].

However, the exact cause of diabetic neuropathy is unknown, while hyperglycemia plays a serious role in the progress of its consequences [7]. The pathophysiology of hyperglycemia which causes microvascular complications in diabetic patients may be explained through several mechanisms [8]. As per Fowler [9], in diabetic rats oxidative stress is thought to be a biochemical activator in cellular injury through decreasing endoneurial blood flow. Russell et al. found that reactive oxygen species can cause neuronal damage when basal glucose levels are slightly altered [10]. Some in vitro and in vivo studies demonstrated that an increase in glucose level leads to the overproduction of oxidative stress biomarkers such as malondialdehyde (MDA), along with the inhibition of endogenous antioxidant synthesis of GSH, SOD, and CAT [11]. Recently, Ostovar et al. have shown that *C. colocynthis* fruit is effective in the treatment of diabetic neuropathy through its antioxidant and hypoglycemic effects [12]. 

The excessive delivery of reactive oxygen species due to oxidative stress also causes apoptosis which is also responsible for diabetic neuropathy. Hyperglycemia causes a latent process called apoptosis, which kills the synapses and neuronal cells in the hippocampus formation [13]. It has been demonstrated that STZ causes diabetes by activating caspase-3 and raising the expression of Bax and Bcl2 in the hippocampus [14]. Chung and colleagues demonstrated that calcimimetics restore diabetic peripheral neuropathy by reducing apoptosis and enhancing autophagy [15].

In recent years, treatment with herbal antioxidants has reduced complications caused by diabetes. *R. cordifolia* belongs to the family *Rubiaceae* commonly known as Manjishtha and Indian Madder generally found in ascending altitudes of the northwestern Himalayas and several hilly areas of India with cylindrical roots—dark-reddish. Traditionally, the roots of *R. cordifolia* are used in the treatment of swelling and inflammation as well as for their antipyretic and analgesic properties [16]. 

The root extract of *R. cordiofolia* possesses an antioxidant effect via inhibiting lipid peroxidation and scavenging free radical formation [17] and also exhibits anti-cancer and antihyperglycemic activity [18]. In the *Chinese Pharmacopeia* [19], *R. cordifolia* is listed for the treatment of several ailments like hematorrhea, dysmenorrhea, arthritis, and diseases associated with free radical generation. One of the studies indicated that the aqueous extract of *R. cordifolia* exhibited antidiarrheal and anti-inflammatory activity in rodents by reducing MDA, IL-1β, and TNF-α levels in colonic tissue [20]. The research showed that an aqueous extract of *R. cordifolia* has an antidiabetic effect in STZ-induced diabetes [18]. It has been reported that *R. cordifolia* along with *Tinospora cordiofolia* ameliorated cell injury by decreasing oxidative stress and exerting neuroprotection during oxygen–glucose deprivation and has an effective therapeutic effect against ischemic brain damage [21].

*R. cordifolia* has some important phytochemicals: alizarin [22], rubiadin [23], manjustin [23], and purpurin [24]. It has been reported that *R. cordifolia* exerts cell/neuroprotective properties and antioxidants against reserpine-induced orofacial dyskinesia [25].

There are various antidepressants and anticonvulsant drugs provided for the management of neuropathic pain approved by the FDA [26]. In today’s scenario, the herbal medicine system is becoming a treatment of interest in a large number of the world population as it can activate several antioxidative processes and protect against oxidative cell damage with little to no negative side effects.

However, research on *R. cordifolia*’s significance in diabetic sequelae, particularly diabetic neuropathy, has not been studied yet. So, the present study’s objective is to examine the neuroprotective effect of a hydroalcoholic extract of *R. cordifolia* roots in STZ-induced diabetic neuropathy in rodents by examining behavioral, oxidative, and apoptotic parameters in sciatic nerve and brain tissues.

## 2. Results

### 2.1. Blood Glucose and Body Weight

**Figure 1:**
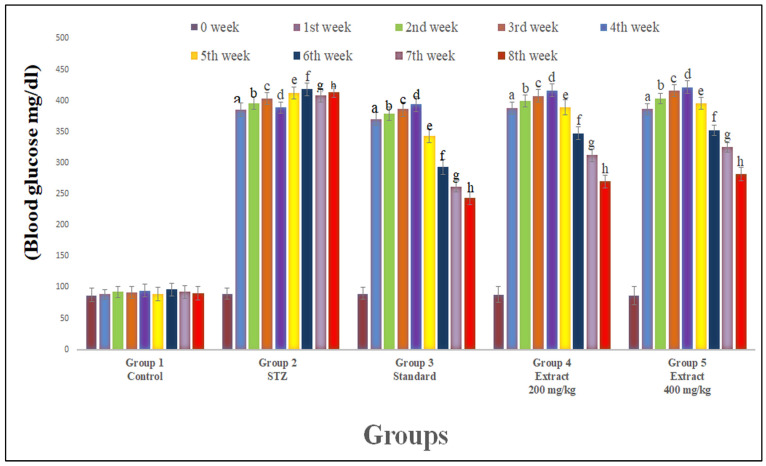
Effect of hydroalcoholic extract of *R. Cordifolia (oral)* on blood glucose level. All data were calculated as the mean ± SD (*n* = 8); data were statistically analyzed using ANOVA and Dunnett’s test. ^a^ *p* < 0.01 compared with group 1 (1st week); ^b^ *p* < 0.01 compared with group 1 (2nd week); ^c^ *p* < 0.01 compared with group 1 (3rd week); ^d^ *p* < 0.01 compared with group 1 (4th week); ^e^ *p* < 0.01 compared with group 1 (5th week); ^f^ *p* < 0.01 compared with group 1 (6th week); ^g^ *p* < 0.01 compared with group 1 (7th week); and ^h^ *p* < 0.01 compared with group 1 (8th week).

After one week of STZ administration, a rise in blood sugar levels was observed compared to the control group (*p* < 0.01). *R. cordifolia* extracts of 200 mg/kg and 400 mg/kg produced significantly decreased serum glucose levels (F_4.35_ = 18.987, *p* < 0.01) compared to the diabetic group in a dose-proportional manner, verify the antihyperglycemic effect of *R. cordifolia* (Figure 1 & Table S1).

**Figure 2:**
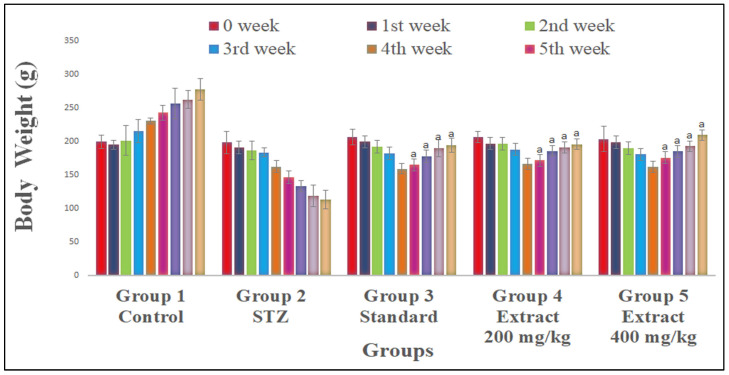
Effect of hydroalcoholic extract of *R. cordifolia* (oral) on body weight. All data were calculated as the mean ± SD (*n* = 8); data were statistically analyzed using ANOVA and Dunnett’s test. ^a^ *p* < 0.01 compared to the negative control group (weeks 5th, 6th, 7th, and 8th).

The significant decrease in bodyweight was examined after the administration of STZ. Body weight increased significantly (F_4.35_ = 39.076, *p* < 0.01) with *R. cordifolia* and glibenclamide compared to the diabetic group. So, it was observed that *R. cordifolia* counteracts the effect of STZ, reflecting its antihyperglycemic activity (Figure 2 & Table S2).

### 2.2. Neuropathic Pain Assessment

#### 2.2.1. Evaluation of Paw Withdrawal Latency (PWL)

**Figure 3:**
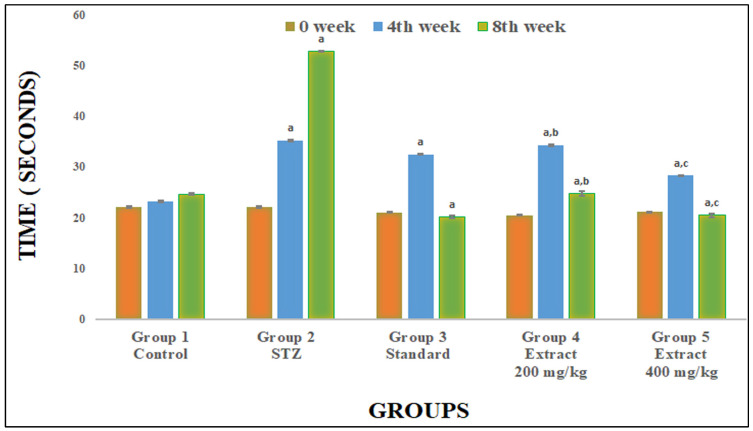
Effect of hydroalcoholic extract of *R. cordifolia (oral)* on thermal latency using the hot plate method. All data were presented as the mean ± SD (*n* = 8); data were statistically analyzed using ANOVA and Dunnett’s test. ^a^ *p* < 0.01 compared with group 1, ^b^ *p* < 0.05 compared with group 2, and ^c^ *p* < 0.05 compared with group 2. At week 0, F(_4.35_) = 30.0767, *p* = 0.00032. At week 4, F(_4.35_) = 1473.819, *p* = 0.00012. At week 8, F(_4.35_) = 2259.85, *p* = 0.00013.

PWL was recorded at 0 days before the administration of STZ and on weeks 4 and 8. *R. cordifolia* (200 mg/kg and 400 mg/kg) and glibenclamide (2.5 mg/kg) significantly increased PWL and inhibited the nociceptive phenomena due to STZ compared to the STZ-induced diabetic group (F_4.35_ = 1473.819, *p* < 0.05) at week 4 and (F_4.35_ = 2259.85, *p* < 0.05) at week 8 (Figure 2). It was found that a higher dose of *R. cordifolia* (400 mg/kg) was more effective towards hot-plate-method-induced algesia than *R. cordifolia* (200 mg/kg) (Figure 3). 

#### 2.2.2. Evaluation of Tail Flick Latency

**Figure 4:**
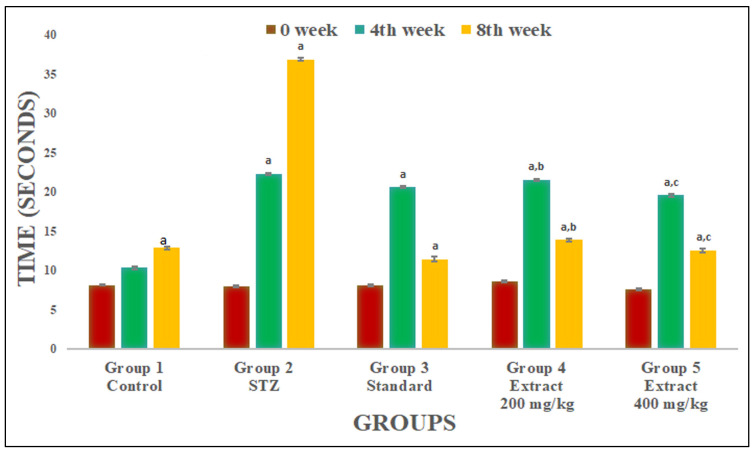
Effect of hydroalcoholic extract of *R. Cordifolia (oral)* on paw withdrawal latency using cold allodynia. All data were calculated as the mean ± SD (*n* = 8); data were statistically analyzed using ANOVA and Dunnett’s test. ^a^ *p* < 0.01 compared with group 1, ^b^ *p* < 0.05 compared with group 2, and ^c^ *p* < 0.05 compared with group 2. At week 0, F(_4.35_) = 9.6181, *p* = 0.00024. At week 4, F(_4.35_) = 1288.956, *p* = 0.00013.

A significant increase in tail flick latency was reported with *R. cordifolia* compared to the STZ-treated diabetic group at week 4 (F_4.35_ = 1288.956, *p* < 0.01) and week 8 (F_4.35_ = 1796.028, *p* < 0.05). Using a 400 mg/kg extract at weeks 4 and 8 showed almost the same result as in the control group and a more effective analgesic effect than a lower dose of extract (200 mg/kg) (Figure 4).

Both methods showed attenuation in pain in a dose-dependent aspect. The difference was statistically significant compared to *R. cordifolia* (200 and 400 mg/kg). However, a higher dose of *R. cordifolia* was more effective towards it. This confirmed that *R. cordifolia* has an analgesic effect, as in previous studies.

### 2.3. R. cordifolia Ameliorates Oxidative Stress

**Figure 5:**
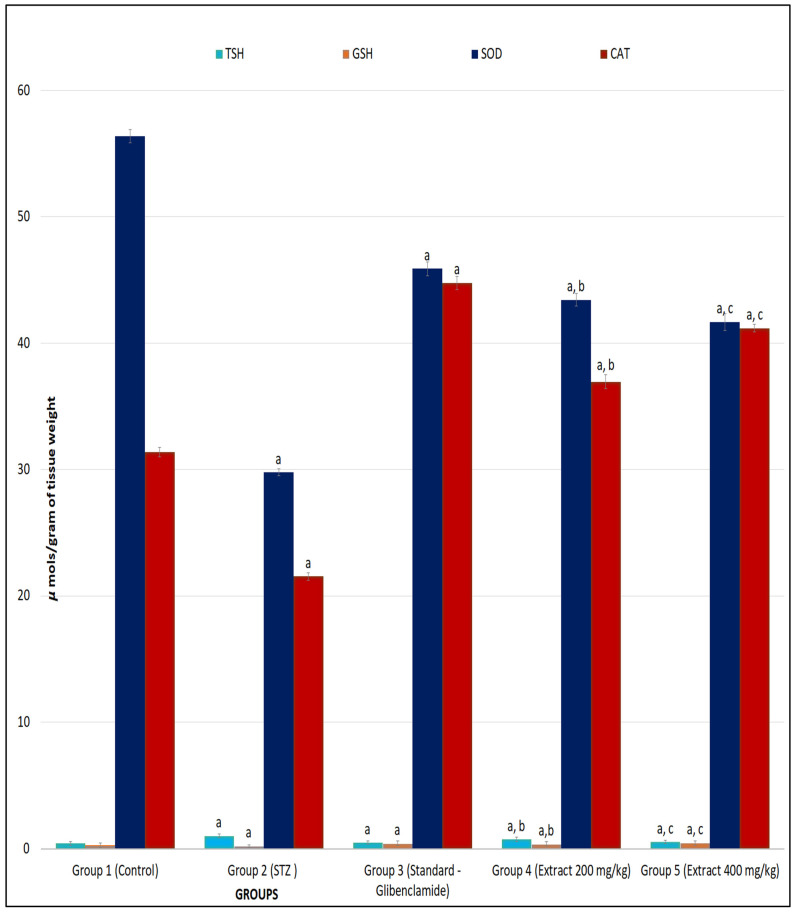
Effect of hydroalcoholic extract of *R. cordifolia (oral)* on lipid peroxidation and antioxidant enzymes on diabetic neuropathy in rat brain tissue. All data were calculated as the mean ± SD (*n* = 8). Data were statistically analyzed using ANOVA and Dunnett’s test. TBARS: ^a^ *p* < 0.01 compared with group 1, ^b^ *p* < 0.01 compared with group 2, and ^c^ *p* < 0.05 compared with group 2. GSH: ^a^ *p* < 0.01 compared with group 1, ^b^ *p* < 0.01 compared with group 2, and ^c^ *p* < 0.05 compared with group 2. SOD: ^a^ *p* < 0.01 compared with group 1, ^b^ *p* < 0.01 compared with group 2, and ^c^ *p* < 0.05 compared with group 2. CAT: ^a^ *p* < 0.01 compared with group 1, ^b^ *p* < 0.01 compared with group 2, and ^c^ *p* < 0.05 compared with group 2.

**Figure 6:**
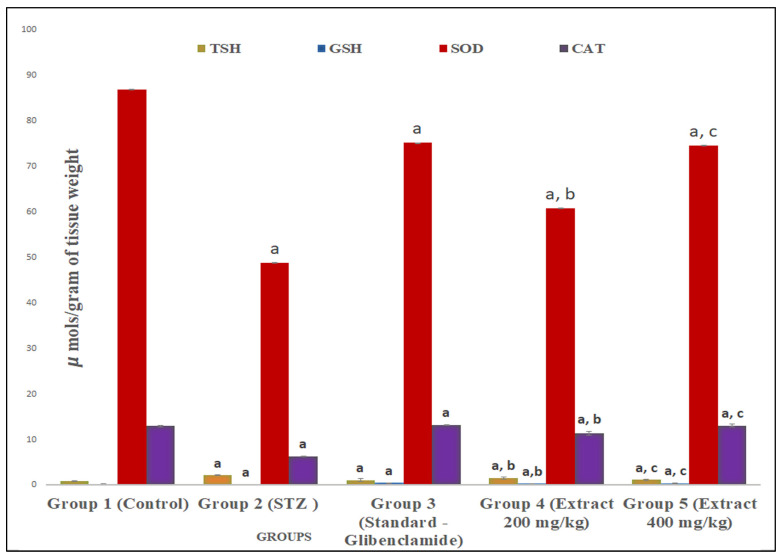
Effect of hydroalcoholic extract of *R. cordifolia (oral)* on lipid peroxidation and antioxidant enzymes on diabetic neuropathy in rat sciatic nerve tissue. All data were calculated as the mean ± SD (*n* = 8). Data were statistically analyzed using ANOVA and Dunnett’s test. TBARS: ^a^ *p* < 0.01 compared with group 1, ^b^ *p* < 0.01 compared with group 2, and ^c^ *p* < 0.05 compared with group 2. GSH: ^a^ *p* < 0.01 compared with group 1, ^b^ *p* < 0.01 compared with group 2, and ^c^ *p* < 0.05 compared with group 2. SOD: ^a^ *p* < 0.01 compared with group 1, ^b^ *p* < 0.01 compared with group 2, and ^c^ *p* < 0.05 compared with group 2. CAT: ^a^ *p* < 0.01 compared with group 1, ^b^ *p* < 0.01 compared with group 2, and ^c^ *p* < 0.05 compared with group 2.

TBARS, GSH, SOD, and CAT levels were measured in brain and sciatic nerve tissues for oxidative stress. In contrast to control group animals, diabetic rats had significantly greater levels of TBARS (*p* < 0.01) and significantly lower levels of GSH (*p* < 0.01) in both tissues of animals. It was found that treatment with *R. cordifolia* (200 and 400 mg/kg/day) in diabetic rats showed a significant (*p* < 0.05 and *p* < 0.01, respectively) decrease in elevated TBARS levels compared to diabetic rats. A total of 400 mg/kg of *R. cordifolia* was more effective than 200 mg/kg compared to diabetic groups (F_4.35_ = 3.1541, *p* < 0.01 in brain) (F_4.35_ = 6.89, *p* < 0.01, in sciatic nerve). This demonstrates that *R. cordifolia* is effective in neuropathic pain and its associated neurological disorders (Figure 5 and Figure 6).

The GSH level significantly (*p* < 0.05 and *p* < 0.001) increased in both tissues of diabetic rats compared to the control group after treatment with *R. cordifolia* (F_4.35_ = 0.3531, *p* < 0.01 in brain; F_4.35_ = 16.1643, *p* < 0.01, in sciatic nerve). This demonstrates protection against the oxidant level in neuropathic pain.

*R. cordifolia* treatment resulted in a highly significant increase in SOD activity when compared to the diabetes control group in both tissues (F_4.35_ = 336.1964, *p* < 0.01 in brain; F_4.35_ = 821.295, *p* < 0.01, in sciatic nerve) that inhibits the excessive release of free radicals in tissues and prevents further damage (Figure 5 and Figure 6).

A significantly high improvement in CAT activity was measured with 400 mg/kg of *R. cordifolia* compared to diabetic animals (F_4.35_ = 469.2894, *p* < 0.01 in brain; F_4.35_ = 6143.8282, *p* < 0.01, in sciatic nerve). It was observed that *R. cordifolia* was equally as effective as the control group. The dose of 200 mg/kg also showed a significant protective effect but it was less than that of 400 mg/kg which showed that the drug is protective against oxidative stress (Figure 5 and Figure 6). So, it was reported that the hydroalcoholic extract of *R. cordifolia* (200 and 400 mg/kg) has an antioxidant effect, as discussed in the previous study.

### 2.4. Effect of R. cordifolia on Apoptosis Factor

**Figure 7:**
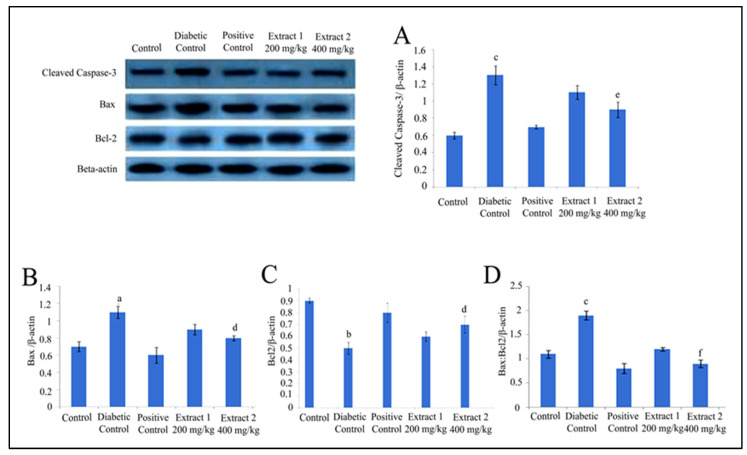
Western blot analysis of rat brain tissue. Western blot analysis of the cleaved caspase-3 (**A**), Bax (**B**), Bcl-2 (**C**), and Bax/Bcl-2 ratio (**D**) protein expression in the brain tissue of rats with STZ administration. Values were expressed as mean ± SD (*n* = 8). Data were statistically analyzed using ANOVA and Dunnett’s test. ^a^ *p* < 0.05, ^b^ *p* < 0.01, and ^c^ *p* < 0.001 versus the control group; ^d^ *p* < 0.05, ^e^ *p* < 0.01, and ^f^ *p* < 0.001 versus the diabetic control group.

**Figure 8:**
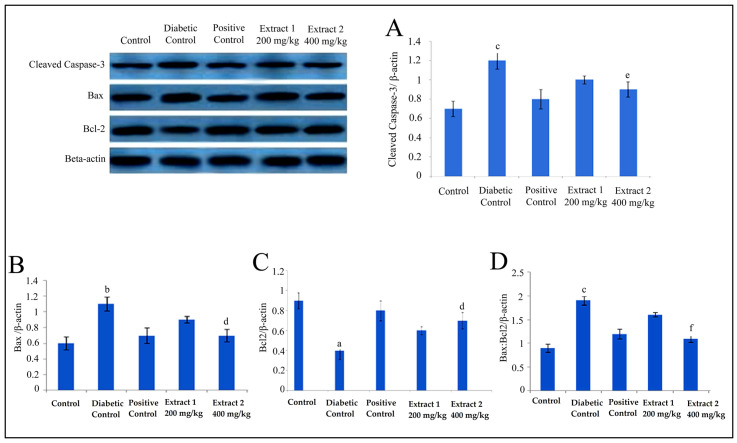
Western blot analysis of rat sciatic nerve. Western blot analysis of the cleaved caspase-3 (**A**), Bax (**B**), Bcl-2 (**C**), Bax/Bcl-2 ratio (**D**), protein expression in the sciatic nerve tissue of rat with STZ administration. Values were expressed as mean ± SD (*n* = 8). Data were statistically analyzed using ANOVA and Dunnett’s test. ^a^ *p* < 0.05, ^b^ *p* < 0.01, and ^c^ *p* < 0.001 versus the control group; ^d^ *p* < 0.05, ^e^ *p* < 0.01, and ^f^ *p* < 0.001 verses diabetic control group.

Western blot data revealed that diabetes causes cleaved caspase-3 activation and Bax protein expression levels in brain and sciatic nerve tissues. In the current study, a significantly increased level of caspase-3 and Bax level were reported in the diabetic group compared to the control group (*p* < 0.05). Additionally, cleaved caspase-3 and Bax protein expression levels in the standard group were lower than in the diabetes group (*p* < 0.05). It was also found that Bcl-2 protein expression level was significantly decreased in diabetic animals compared to the control group (*p* < 0.05). The treatment with *R. cordifolia* (200 and 400 mg/kg) inhibited the activation of caspase-3, increased the Bcl-2 protein expression level, and decreased the Bax level in the brain and sciatic nerve tissues compared to the diabetic group (*p* < 0.05). Thus, the significant decrease in the Bax:Bcl-2 ratio in brain and sciatic nerve tissues in *R. cordifolia* treated groups was measured compared to the diabetic group (*p* < 0.001) (Figure 7 and Figure 8).

### 2.5. Histopathological Evaluation

**Figure 9:**
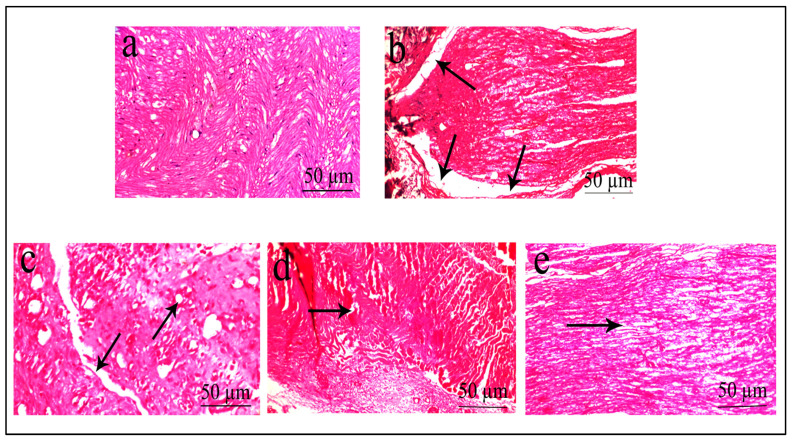
Histopathological study of the sciatic nerve. Histopathological examination of sciatic nerve tissue (image at 40×, stained with hematoxylin and eosin). (**a**) Control group: no swelling, vocalization, or degradation of myelin sheath and axon; (**b**) Demonstrates vacuolar degeneration with swelling along with axonal degeneration, with fraying of fibers (arrow) in STZ-treated rats; (**c**) The sciatic nerve showed mildly degenerated axons and edema in the positive control group; (**d**) Less vocalization and dispersion of fibers with the smaller diameter with extract (200 mg/kg) as compared to STZ-treated group; (**e**) No swelling and vacuolization was mild or needed no degradation of myelin sheath via extract (400 mg/kg) compared to STZ-treated group.

The sciatic nerve’s histopathology revealed nearly normal, uniformly spaced axons within its myelin sheath, with no signs of inflammatory infiltration, degenerative alterations, or any other abnormalities in the control group (Figure 9a). The diabetic group displayed axonal loss and degeneration of axons with many regenerating thin myelin axons and few degraded bodies (Figure 9b). *R. cordifolia* (200 mg/kg) showed almost non-inflammatory infiltration and mild or no degeneration of the myelin sheath (Figure 9d). A high dose of *R. cordifolia* (400 mg/kg) revealed minor axonal degenerative changes without regenerative features (Figure 9e). The histopathology changes of tissues treated with *R. cordifolia* demonstrated that it was effective in treating diabetic neuropathy.

**Figure 10:**
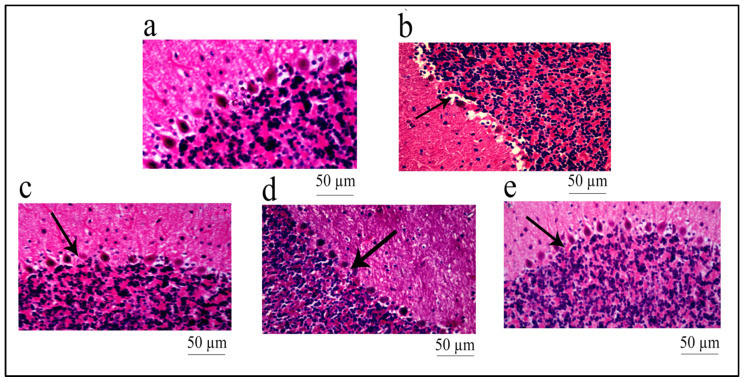
Histopathological study of the brain (cerebellum) tissue. Histopathological examination of brain tissue (cerebellum) (images at 40×, stained with hematoxylin and eosin. (**a**) Control group: no edematous and degenerative tissue and no change in architecture; (**b**) Demonstrates vacuolar degeneration with edematous tissues and architectural loss (arrow) of neurons in STZ-treated group; (**c**) Cerebellar area showed less edema and vacuolization in the positive group; (**d**) Less vacuolization and dispersion of fiber with similar diameters with the lower dose (200 mg/kg) of extract as compared to STZ-treated group; (**e**) No swelling and mild vacuolization or no degeneration of myelin sheath with the higher dose (400 mg/kg) of the extract were observed compared to STZ-treated group.

Histopathological examination of the brain tissue (cerebellum) showed no change in the architecture as well as no edema or degeneration in tissues in the control group (Figure 10a). The diabetic group demonstrated vascular degeneration with edematous tissues and architectural loss of neurons in the tissues (Figure 10b). The treatment with *R. cordifolia* (200 mg/kg) (Figure 10d) showed less vacuolization and dispersion of fibers with a similar diameter compared to the diabetic group, whereas treatment with *R. cordifolia* (400 mg/kg) (Figure 10e) showed no swelling and mild vacuolization or no degeneration of the myelin sheath compared to the diabetic group.

## 3. Discussion

Diabetes mellitus is associated with diabetic neuropathy, a neurodegenerative condition that can impact the somatic as well as autonomic peripheral nerve systems. STZ-induced diabetic rats are used to study diabetic neuropathy with diabetic symptoms such as hyperalgesia and allodynia [27].The altered nociception pattern is not due to STZ’s neurotoxicity but rather its hyperglycemia-induced pathophysiological symptoms [28]. Numerous studies, including the one we carried out, revealed that diabetic rats significantly delayed their paw and tail withdrawal latencies in comparison to control animals [29]. This study reported the protective effect of *R. cordifolia* in a rodent model of diabetic neuropathy induced by STZ. This study showed that administration of a hydro-alcoholic extract of *R. cordifolia* for 4 weeks significantly reduced hyperalgesia (both hot and cold), increased both paw and tail withdrawal latencies, significantly improved oxidant levels, reduced nerve degeneration, and decreased the apoptotic marker Bax/Bcl-2 ratio and caspase-3 activation in the sciatic nerve and brain tissues, concluding that *R. cordifolia* modulated the neuropathic pain induced by high glucose levels.

Numerous researches have demonstrated that *R. cordifolia* possesses immunosuppressive, analgesic, anti-inflammatory, anti-oxidative, anti-tumor, and neuroprotective properties [30]. It has also been reported that an increase in glucose levels leads to diabetic neuropathy which can cause damage to the nerve tissues and ultimately leads to neuropathic pain which is the classic complication of diabetes [31]. The current study reported the blood glucose level in Wistar rats was significantly decreased and the weight of the animals was also found to be significantly increased from the first week to eight weeks after starting the treatment with *R. cordifolia* incrementally.

The analgesic and anti-inflammatory effect of *R. cordifolia* is consistent with the previous studies [32]. Diwane et al. [33] also reported that *R. cordifolia* ameliorated the paclitaxel-induced neuropathic pain and increased the paw latency. In our study, a significant increase in paw and tail withdrawal latency was found in STZ-induced neuropathy with *R. cordifolia* (200 & 400 mg/kg) administered dose-dependently as compared to diabetic rats.

Oxidative stress is one of the key factors contributing to diabetic neuropathy [34], and it is mostly associated with the oxidation of proteins and monosaccharides [35]. A useful in vivo paradigm for the investigation of diabetic neuropathy is the hyperglycemia-induced cell death in STZ-induced diabetic rats [36]. It was previously reported by our team that severe oxidative stress directly damages nerve tissue [37]. Several exploratory diabetes models have been used by scientists to examine the effects of different antioxidant-based drugs on redox profiles as a result of this understanding. Furthermore, it is widely known that oxidative stress causes apoptosis in several different cell types [38].

Apoptosis can result from several pathogenic mechanisms, such as the disturbance of intracellular redox balance and irreversible oxidative changes of lipids, proteins, or DNA. As a result of oxidative stress, apoptosis also contributes significantly to the onset of diabetic neuropathy [39]. Numerous in vivo and in vitro studies have revealed that, in contrast to the normal state, hyperglycemia increases the expression of the pro-apoptotic Bax protein (increased Bax:Bcl2 ratio) [40]. In a recent study, it was discovered that rosemaric extract prevented hyperglycemia-induced cellular damage by lowering the levels of various biochemical indicators of apoptosis, such as caspase-3 activation and the Bax/Bcl2 ratio, which was similar to the results of our investigation [41].

The results of this investigation demonstrated that *R. cordifolia* possesses antioxidant properties that are demonstrated by its capacity to reduce the increase in MDA levels in tissue homogenate. Additionally, it reversed the reduction of GSH levels in both tissues in a dose-related manner compared to a diabetic group. Likewise, *R. cordifolia* reversed the STZ effect on SOD and CAT activity in both doses and was able to activate SOD and CAT levels that were close to normal. According to the evidence, hyperglycemia in STZ-induced diabetic neuropathy causes severe molecular abnormalities, including an exceptional activation of the oxidative phosphorylation pathway in mitochondria and changes in the way lipids are metabolized in tissues. It is also reported in the present study that lowering the expression of Bax and the ratio between Bax/BCl2 with the treatment of *R. cordifolia* reverses diabetic neuropathy in rats. This confirmed the apoptosis effect of the Bax/Bcl-2 ratio and caspase-3 in the diabetic neuropathy rats.

An in vitro study reported that inhibition of apoptotic proteins by Z-VAD-FMK (as a caspase inhibitor) blocks cell injury caused by high glucose levels [42]. According to researchers, animals with peripheral neuropathy exhibit pain-related behavior that is influenced by caspases, which are part of the apoptosis signaling pathways. Some animal studies suggest that axonal degeneration following peripheral nerve injury induces neuropathy, even if all of the mechanisms behind diabetic neuropathy are not well understood [43].

In light of this information, researchers have examined the impact of several antioxidant-based therapies on redox status in several exploratory models of diabetes. Recently, herbal polyphenolic substances have received a lot of interest because they can activate several antioxidative pathways and induce protective enzymes, which protect cells from oxidative damage [44]. Generally, the root, leaves, fruits, stem, etc., of *R. cordifolia* are used for their medicinal properties such as analgesic and anti-inflammatory activities [45]. *R. cordifolia* also has several important biological activities, such as hepatoprotective [46], antidiabetic [47], anti-oxidant [48], immunomodulatory [49], and neuroprotective effects [21]. According to various sources, *R. cordifolia* has potent antioxidant properties, and studies on its extract have revealed that it has considerable anti-radical properties. In light of this, both the cold allodynia and hot plate method of drug-treating rats with an alcoholic extract of the roots and rhizomes of *R. cordifolia* significantly decreased the withdrawal latency in paclitaxel-induced neuropathic pain in rats. The outcomes could be a result of GABA’s involvement or an antioxidant mechanism [33]. Additionally, *R. cordifolia* has been shown to effectively treat ischemia-reperfusion injury by decreasing the extent of myocardial infarction, inhibiting apoptosis, autophagy, and inflammation, and preserving the structural and functional integrity of myocardial and endothelial cells [50].

*R. cordifolia’s* dual analgesic and anti-inflammatory effects might be explained by the reduction in neuronal death. The neuroprotective effects of *R. cordifolia* may be attributable to the plant’s antioxidant, anti-inflammatory, or free-radical-scavenging properties due to the presence of some important phytochemicals such as flavones, flavonoids, anthraquinones, and free anthraquinones, which are generally found in the root extract of *R. cordifolia* [50,51]. The antioxidant effect might be due to the flavonoids and anthraquinones present in the extract, which is in good concord to in vitro and in vivo studies carried out in this work.

## 4. Materials and Methods

### 4.1. Chemicals

The chemicals were procured from CDH, Delhi, India. STZ was purchased from UVSAR, Ghaziabad, India and glibenclamide was purchased from Medico Remedies Ltd., Mumbai, India.

### 4.2. Plant Authentication

*R. cordifolia* was collected and authenticated from Raw Materials Herbarium and Museum, Delhi, India, authentication number NISCAIR/RHMD/consult/2019/3510-11 for further reference.

### 4.3. Preparation of Plant Extract

*R. cordifolia’s* roots were dried at room temperature and ground into coarse powder with the help of a high-speed electric grinder. The powdered drug is defatted by using petroleum ether as a solvent in a soxhlet apparatus. Then Marc is used for successive soxhalation (24 h) to obtain hydroalcoholic (40:60 *v*/*v* ethanol in water) extract at 75–79 °C [52]. The extract was concentrated through a rotary evaporator (The Rotavapor^®^ R-100, Buchi, Flawil, Switzerland) followed by complete drying in a vacuum dryer (VTD-12, Shree Bhagwati Machtech India Pvt. Ltd., Ahmedabad, India); the dried extract was kept in a refrigerator for further use.

### 4.4. Experimental Animals

Eight Wistar rats per cage were used to house the forty adult male rats, which ranged in weight from 150 to 300 g. The animals were fed and watered and kept at 23 ± 2 °C and 55% humidity during a natural 12 h cycle. To prevent unnecessary stress, animals were acclimated to the experimental environment before the study’s start date for one week. The Institutional Animal Ethical Committee of I.T.S. College of Pharmacy, Ghaziabad (1044/PO/Re/S/07/CPCSEA) has properly approved the current study’s animal procedures.

### 4.5. Drug Administration and Group Design

STZ, at a concentration of 60 mg/kg dissolved in 0.1 M citrate buffer (0.1 M, pH 7.4), was administered (i.p.) to rats after an overnight fast. A commercial glucometer (Dr. Morepen BG-03, Morepen Laboratories Limited, New Delhi, India) and test strips were used to monitor blood glucose levels 48 h after STZ treatment to confirm the presence of diabetes [53]. Rats with plasma glucose values greater than 250 mg/dL were selected for the study [54]. Each group had 8 animals and was divided into the following groups:

Group 1 (i.e., control group): 0.25% CMC in saline was administered for 8 weeks.

Group 2 (i.e., diabetic group): Administered a single dose of STZ (60 mg/kg; i.p.) on the first day of the study.

Group 3 (i.e., positive control group): Administered glibenclamide (2.5 mg/kg from the 5th week to the 8th week) and STZ (single dose of 60 mg/kg on the first day of study).

Groups 4 and 5 (i.e., test groups) concurrently received *R. cordifolia* (200 and 400 mg/kg; p.o) dissolved in 5% DMSO in normal saline, i.e., NaCl 0.9%/DMSO (95%/5%, *v*/*v*) from the 5th week to the 8th week and STZ (single dose of 60 mg/kg on the first day of study), respectively.

During the 0th, 4th, and 8th weeks, cold allodynia and hot plate methods were performed for the measurement of tail flick and paw withdrawal latency. The animals’ blood glucose levels and body weight were measured every week. The beginning of drug therapies occurred between the fifth and eighth weeks [53]. Animals were anesthetized using an intraperitoneal injection of thiopental sodium (50 mg/kg) and sacrificed at the end of the study after the assessment of behavioral parameters. The sciatic nerve and brain tissues were collected for biochemical histopathological and Western blot examination.

### 4.6. Blood Glucose Level

Blood sugar levels were measured before and after streptozotocin injection at the start of the study. Every week, blood glucose levels of specific animals were measured up to the 8th week of the study.

### 4.7. Body Weight

The body weight was measured every week till the end of the study.

### 4.8. Behavioural Experiments

#### 4.8.1. Tail Flick Latency

The old allodynia method was used to evaluate the tail flick latency. The animal’s tail’s dorsum was kept in a cup of 10 °C cold water and time taken to flick its tail to avoid the painful stimulation was recorded. The cutoff time of 15 s was to be considered to prevent any injury. This was performed on the 0th, 4th, and 8th week of the study before sacrificing the animal [55].

#### 4.8.2. Hot Plate Method

A hot plate at 55 °C was used to measure the hyperalgesic response to thermal nociceptive stimuli on the 0th, 4th, and, 8th week of the study. The time taken by an animal to lick its paws for the first time or jump to protect itself from heat was used to assess the pain threshold [56].

### 4.9. Biochemical Estimation

#### 4.9.1. TBARS Level

TBARS analysis was used to measure the level of lipid peroxidation in tissue supernatants (brain and sciatic nerve). A total of 1 mL of a supernatant solution of 10% tissue homogenate was added to 1 mL of 30% TCA and 8% TBA reagent. All test tubes had aluminum foil sheets covering them, and they were agitated in the water bath for 30 min at 80 °C and then the solution for 30 min. At 535 nm, the supernatant solution absorbance was measured in comparison to a blank solution and demonstrated as n moles MDA/mg protein [57].

#### 4.9.2. GSH Level

To 2 mL of 10% homogenate, 2.5 mL EDTA (0.02 M) was added. A total of 4 mL of cold distilled water was added to the above solution. The mixture was thoroughly agitated for 10 min while being combined with one milliliter of 50% TCA. After 10 min, the solution was centrifuged for 15 min. A total of 4 mL of Tris buffer (0.4 M, pH 8.9) was mixed with 2 mL of supernatant and the absorbance at 412 nm was recorded and expressed as µM GSH/mg protein after adding DTNB within 5 min against a blank solution, made up of 2 mL of distilled water without homogenate [58].

#### 4.9.3. CAT Activity

Catalase activity in tissue supernatant was determined by H_2_O_2_ used per min/mg of protein. First, the absorbance of H_2_O_2_ increases with a decrease in the UV range. The catalase activity was measured as the difference in absorbance per unit time. A total of 10% of tissue homogenate of all animals in each group (*n* = 8) was poured into phosphate buffer solution (50 mM, pH 7.4) and centrifuged at 3000 rpm for 15 min. A total of 2.95 mL of 19 mM H_2_O_2_ were mixed with 50 µL supernatant. The change in absorbance levels was recorded at 240 nm for 3 min at an interval of 1 min [59].

#### 4.9.4. SOD Level

Superoxide dismutase activity in tissues was measured by adding Tris HCL buffer to 100 µL of cytosolic supernatant. The absorbance was noted at 420 nm after the addition of pyrogallol and demonstrated as units/mg protein [60].

### 4.10. Western Blot Analysis

The samples were prepared and the sections were blocked in 10% normal goat serum at room temperature for 1 h. The cleaved Bax/Bcl-2 rabbit monoclonal antibody (1:200, Cell Signaling Technology Inc., Danvers, MA, USA) was diluted, which recognizes endogenous levels of the large fragment of activated Bax, and Bcl-2 rabbit monoclonal antibody in the recommended antibody diluents was then added to each section and incubated at 4 °C overnight. The sections were rinsed three times in PBS and incubated using a biotinylated goat anti-rabbit secondary antibody (1:2000) at room temperature for 1 h, then incubated using avidin–biotin–peroxidase solution (ABC reagent) for 30 min at room temperature, and washed three times in PBS. Then, the samples were treated with DAB for 2 min. Finally, the sections with hematoxylin that were dehydrated and cover slipped were counterstained. The immunohistochemical staining of caspase-3 was the same as the Bax and Bcl-2. RIPA lysis buffer was used to extract the total protein. Then, the protein concentration was estimated by the method of Coomassie Brilliant Blue. Subsequently, 40 μg proteins per lane were loaded into 10% SDS-PAGE gel for electrophoresis, followed by the transfer to polyvinylidene difluoride membrane (PVDF) (Millipore Co., Bedford, MA, USA) which was incubated overnight at 4℃ temperature and 7.4 pH in blocking buffer. Membrane washing was performed thrice using washing buffer (20 mM Tris–HCl, 150 mM NaCl, 0.1% Tween 20) and blots were probed with rabbit polyclonal anti-Bax, anti-caspase-3, and anti-Bcl 2 antibodies for approximately 3 h. They were subsequently washed with buffer and probed with horseradish peroxidase-conjugated with goat anti-rabbit secondary antibody for 1 h.

Blots were incubated by HRP-conjugated secondary antibody and bands were developed using enhanced chemiluminiscence (Tiangen Co., Beijing, China). The β-actin was used as reference for normalization [61].

### 4.11. Histopathological Evaluation

The tissues were preserved in formalin solution (10% *v*/*v*) and rinsed in PBS. They were stained with hematoxylin and eosin (H&E) for histopathological study.

### 4.12. Statistical Analysis

The results were calculated as mean ± SD. Data were statistically analyzed using ANOVA and Dunnett’s test by using SPSS software. It was statistically significant when the *p*-value was found to be <0.05.

## 5. Conclusions

In conclusion, the findings of this study demonstrate the potential therapeutic benefits of *R. cordifolia* in the management of diabetic neuropathy. The experimental evidence presented here suggests that *R. cordifolia* exerts its effects through multiple mechanisms, including antidiabetic, analgesic, antioxidant, and anti-apoptotic actions. These effects were observed in a Wistar rat model of diabetic neuropathy, where *R. cordifolia* treatment led to improvements in pain thresholds, reduced oxidative stress, lowered blood glucose levels, and increased body weight. Furthermore, the histopathological analysis revealed a significant improvement in the structural integrity of the brain and sciatic nerve tissues, particularly at a higher dose of *R. cordifolia* (400 mg/kg). This suggests that *R. cordifolia* may play a crucial role in preserving the architecture and preventing axonal degeneration associated with diabetic neuropathy. The downregulation of cleaved caspase-3 and the reduction in the Bax:Bcl2 ratio in sciatic nerve and brain tissues further highlight the potential anti-apoptotic properties of *R. cordifolia*. Overall, the results of this study support the idea that *R. cordifolia* has promising neuroprotective, antidiabetic, and analgesic properties, making it a potential candidate for further exploration as a therapeutic agent in the management of diabetic neuropathy. Further research is needed to fully elucidate the underlying molecular mechanisms and to validate these findings in clinical settings.

## Data Availability

Data are contained within the article.

## Supplementary Materials

The following supporting information can be downloaded at: https://www.mdpi.com/article/10.3390/ph16111586/s1, Tabe S1: Effect of hydroalcoholic extract of *R. cordifolia* on Blood Glucose Level, Table S2; Effect of hydroalcoholic extract of *R. cordifolia* on Body weight.
