# Supplementary material for: Rubia cordifolia L. Attenuates Diabetic Neuropathy by Inhibiting Apoptosis and Oxidative Stress in Rats
Source: Pharmaceuticals (Basel). 2023 Nov 9;16(11):1586. doi: 10.3390/ph16111586 (PMC10674165; doi:10.3390/ph16111586)
Supplement: Supplementary file 1 [file pharmaceuticals-16-01586-s001.zip › pharmaceuticals-2634439-supplementary.pdf]

Table S1 : Effect of hydroalcoholic extract of *R. cordifolia* on Blood Glucose Level

Groups	0 week	Week 1	Week 2	Week 3	Week 4	Week 5	Week 6	Week 7	Week 8
**Group 1 Control**	**87.76±11.4**	**88.65±7.97**	**92.76±8.98**	**91.65±9.87**	**94.56±9.99**	**89.34±10.54**	**96.25±10.44**	**92.34±10.33**	**90.45±10.54**
**Group 2 STZ**	**89.8±9.38**	**385.45±11.44**[a]	**396.33±10.44**[b]	**403.32±9.55**[c]	**388.76±8.87**[d]	**412.65±9.76**[e]	**418.33±9.76**[f]	**408.43±10.44**[g]	**414.22±9.11**[h]
**Group 3 Standard**	**90.2±9.79**	**370.33±10.33**[a]	**379.34±10.83**[b]	**386.43±11.76**[c]	**394.43±11.77**[d]	**343.22±10.43**[e]	**293.11±12.32**[f]	**262.11±9.21**[g]	**243.34±9.99**[h]
**Group 4 Extract 200mg/kg**	**88.6±12.84**	**388.45±9.65**[a]	**399.34±9.87**[b]	**407.54±10.34**[c]	**416.76±10.45**[d]	**389.23±12.45**[e]	**347.43±10.34**[f]	**312.21±9.83**[g]	**269.98± 9.73**[h]
**Group 5 Extract 400mg/kg**	**86.9±14.89**	**386.54±9.1**[a]	**403.65±8.65**[b]	**415.76±9.8**[c]	**421.69±10.23**[d]	**395.46±9.38**[e]	**352.35±8.6**[f]	**324.87±7.87**[g]	**281.62±10.76**[h]

Effect of hydroalcoholic extract of *R. cordifolia* on Blood Glucose Level. All data were calculated as the mean ± SD (n=8); [a]p<0.01 compared with group 1 (1st week); [b]p <0.01 compared with group 1 (2nd week); [c]p<0.01 compared with group 1 (3rd week); [d]p<0.01 compared with group 1(4th week); [e]p<0.01 compared with group 1 (5th week); [f]p<0.01 compared with group 1 (6th week); [g]p<0.01 compared with group 1 (7th week); [h]p<0.01 compared with group 1 (8th week).

Table S2: Effect of hydroalcoholic extract of *R. cordifolia* on Body weight

Groups	0 week	Week 1	Week 2	Week 3	Week 4	Week 5	Week 6	Week 7	Week 8
Group 1 Control	199.67±9.95	194.8±6.75	201.3±22.6	215.7±17.3	230.4±4.61	242.7±11.15	256.8±22.7	262.4±13.62	277.5±15.72
Group 2 STZ	198.29±16.45	190.6±9.47	186.46±13.83	183.58±6.35	162.42±8.5	146.42±9.38	132.89±8.24	118.46±15.8	112.79±13.73
Group 3 Standard	206.89±11.6	199.67±8.88	191.85±9.78	181.99±8.99	158.89±7.75	164.87±8.98 [a]	177.66±9.65 [a]	189.54±12.76 [a]	194.52±10.75 [a]
Group 4 Extract 200mg/kg	205.97±8.34	196.76±8.98	196.75±9.67	187.88±9.1	166.43±7.99	171.54±8.65 [a]	185.65±7.88 [a]	191.22±8.43 [a]	195.75±7.5 [a]
Group 5 Extract 400mg/kg	203.56±18.49	198.65±9.87	189.76±9.12	180.44±8.67	162.39±8.41	175.65±8.98 [a]	184.79±8.65 [a]	192.8±7.87 [a]	209.31±7.55 [a]

Effect of hydroalcoholic extract of *R. cordifolia* on Body weight. All data were calculated as the mean ± SD (n=8); [a]p<0.01 compared to the negative control group (weeks 5th 6th 7th 8th).
